# The applications of machine learning in plastic and reconstructive surgery: protocol of a systematic review

**DOI:** 10.1186/s13643-020-01304-x

**Published:** 2020-02-28

**Authors:** Angelos Mantelakis, Ankur Khajuria

**Affiliations:** 1 Lewisham and Greenwich NHS Trust, London, UK; 2 London, UK; 3 Kellogg College, University of Oxford, Oxford, UK; 4 Department of Surgery and Cancer, Imperial College London, London, UK

**Keywords:** Artificial intelligence, Machine learning, Deep learning, Plastic surgery, Big data

## Abstract

**Background:**

Machine learning, a subset of artificial intelligence, is a set of models and methods that can automatically detect patterns in vast amounts of data, extract information and use it to perform various kinds of decision-making under uncertain conditions. This can assist surgeons in clinical decision-making by identifying patient cohorts that will benefit from surgery prior to treatment. The aim of this review is to evaluate the applications of machine learning in plastic and reconstructive surgery.

**Methods:**

A literature review will be undertaken of EMBASE, MEDLINE and CENTRAL (1990 up to September 2019) to identify studies relevant for the review. Studies in which machine learning has been employed in the clinical setting of plastic surgery will be included. Primary outcomes will be the evaluation of the accuracy of machine learning models in predicting a clinical diagnosis and post-surgical outcomes. Secondary outcomes will include a cost analysis of those models. This protocol has been prepared using the Preferred Items for Systematic Review and Meta-Analysis Protocols (PRISMA-P) guidelines.

**Discussion:**

This will be the first systematic review in available literature that summarises the published work on the applications of machine learning in plastic surgery. Our findings will provide the basis of future research in developing artificial intelligence interventions in the specialty.

**Systematic review registration:**

PROSPERO CRD42019140924

## Background

In the era of big data, the plethora of efforts towards gathering and analysing patient data in large scale is rapidly increasing [1]. Amongst others, these efforts try to improve the diagnosis of diseases and the prediction of post-treatment outcomes using large amounts of data from past cases. The analysis of this vast amount of information, however, is beyond the capabilities of traditional statistical methods previously used in academic medicine [2].

Machine learning, a subset of artificial intelligence, is the set of models and methods that can automatically detect patterns in vast amounts of data, extract information and use it to perform various kinds of decision-making under uncertain conditions [3]. These models have the potential of two principally distinct functions: supervised and unsupervised learning (termed “deep learning”). Supervised learning involves the creation and optimisation of statistical models which aim to predict an outcome using information from past cases [2, 4]. In contrast, unsupervised learning aims to identify patterns in previously seemingly random data and generate novel associations [2, 4, 5].

Healthcare professionals have been quick to adopt these emerging technologies to improve patient outcomes [5]. Examples include machine learning models created to identify clinical diagnoses, which perform to the level of expert clinicians in identifying acute cerebral ischaemia, malignant skin lesions and lung cancer subtypes [6–8]. In the field of surgery, this technology has demonstrated a unique potential in predictive post-operative success and complication rate in procedures such as traumatic brain injury, cervical spine fusion and glioma removal, amongst others [9–11].

This technology has the potential to provide clinically relevant information across many areas of plastic surgery. In burn surgery, machine learning has been used to predict whether complete wound healing will require more or less than 14 days with an accuracy rate of 86% [12]. In the field of microsurgery, authors have been able to predict surgical site infections following free flap reconstruction in head and neck cancer with a sensitivity of 81% and specificity of 61% through using artificial intelligence neural networks [13]. Further, machine learning has also been applied in aesthetic surgery research, where using supervised learning, the authors were able to extract potential beauty-determining facial features to guide pre-operative planning [14].

The aim of this review is to systematically analyse the available literature on the applications of deep learning in plastic surgery. Data collected will be used to provide an up-to-date overview of the potential utility of this technology in the specialty and suggest future directions of further research.

## Methods

### Aim

This systematic review is intended to evaluate the clinical applications of machine learning models in the field of plastic and reconstructive surgery and to determine areas of future research on this technology.

### Protocol and registration

This protocol is registered in the Prospective Register of Ongoing Systematic Reviews (PROSPERO) CRD42019140924 and adheres to the Preferred Reporting Items for Systematic Review guidelines and Meta-Analysis Protocols (PRISMA-P 2015) [15] [Additional File].

### Search strategies

All studies published between 1990 and the date of the search will be considered for review.

We will perform a comprehensive search of MEDLINE (OVID SP), EMBASE (OVID SP), Science Citation Index, ClinicalTrials.gov and CENTRAL. A combination of free text and Medical Subject Headings (MeSH) terms will be used. An example search strategy in MEDLINE is the following:

**Table:** 

1	(“deep learning” OR “artificial intelligence” OR “machine learning” OR “decision trees” OR “random forests” OR SVM OR “support vector machine”)
2	exp “NEURAL NETWORKS (COMPUTER)”/ OR exp “DEEP LEARNING”/
3	exp “ARTIFICIAL INTELLIGENCE”/
4	(1 OR 2 OR 3)
5	(microsurgery OR (surgery AND (plastic OR reconstructive OR esthetic OR aesthetic OR burns OR hand OR craniofacial OR “peripheral nerve”)))
6	exp “SURGERY, PLASTIC”/ OR exp “RECONSTRUCTIVE SURGICAL PROCEDURES”/
7	(5 OR 6)
8	(4 AND 7)

### Identification and selection of studies

Following database searching, studies will be populated into Endnote X7 library (Clarivate Analytics, USA). There will be two stages of screening, carried by two independent reviewers using pre-specified criteria. The search results, including abstracts, full-text articles and record of reviewer’s decisions, including reasons for exclusion, will be recorded.

1. Stage 1: Title and abstract review. This will be carried out by the two independent researchers by adhering to the set eligibility criteria. Any discrepancies will be resolved through a consult by a third reviewer.
2. Stage 2: Studies included will undergo full-text review by the same independent reviewers. Any discrepancies will be resolved through a consult to a third reviewer.

### Eligibility criteria

#### Types of studies

Any primary studies (including case reports), which assess the prediction rate of deep learning models in diagnosis of disease or post-operative outcomes in plastic surgery, either on its own or compared to other techniques, will be included. There will be no geographical restriction. Our exclusion criteria include studies utilising machine learning without clinical data, non-English language articles and review articles.

#### Types of study participants

We will include clinical data from adult participants (> 18 years old) with conditions requiring plastic or reconstructive surgery. Data from animal studies will be excluded.

#### Types of interventions

The studies considered will present artificial intelligence models utilising deep learning as an intervention with the aim to provide a diagnosis of a clinical presentation, or a clinical prognosis of a plastic surgery intervention. The intervention may be used by itself or in combination with other methods. Since this technology is new, there is no single best deep learning model, and because of the versatility of conditions treated in plastic surgery, it is expected that various different models will be identified in our review.

### Outcomes

#### Primary outcomes

The primary outcomes will be the evaluation of deep learning models on two distinct functions. The first function is the accuracy of providing a clinical diagnosis. Studies must have a defined clinical condition for which the model is designed to identify. The accuracy of performing this task (either on its own or in assistance with a clinician) will be collected.

The second primary outcome will be the accuracy of prediction of post-operative outcomes and complications of plastic surgery interventions. In order to qualify, studies will need to have created a model to predict a particular clinical outcome (for example, probability of post-operative wound infection), with data collected prospectively or retrospectively.

In both settings, the model’s accuracy will be assessed by the reported specificity, sensitivity, positive predictive value and negative predictive value of performing the named task.

#### Secondary outcomes

The secondary outcomes will include cost analysis of the deep learning models. Further, outcomes of studies that have utilised deep learning models as a treatment for a clinical condition (for example, neuroprosthesis) will also be collected.

### Data extraction, collection and management

After the study selection is completed, the two reviewers will independently extract data using a standardised data extraction form. Any disagreements and differences will be resolved by discussion with a third reviewer.

The following data will be extracted:

1. Study characteristics (authors, year of publication, study design)
2. Patient demographics (number of participants, sex, mean age)
3. Indication of application of the software model (prediction of a diagnosis or treatment outcome)
4. Software characteristics
5. Outcomes (specificity, sensitivity, positive predictive value and negative predictive value of forming a diagnosis; predicting rates of overall survival, treatment success, post-operative function, aesthetic outcome, complications and recurrence)
6. Complications or adverse events reported

### Risk of bias

The risk of bias in the selected randomised controlled trials will be evaluated by the two independent reviewers through utilising the Cochrane Collaboration Risk of Bias tool [16]. The methodological quality will be assessed based on appropriate participant selection and randomisation, blinding of participants and reviewers, attrition, selective reporting and others. An overall grading of low, medium or high risk of bias will then be allocated. For non-randomised trials, the ROBINS-I (Risk of Bias in Non-randomised Studies-of Interventions) will be utilised [17]. For quantitative studies in which the ROBINS-I is not applicable, risk of bias assessment will be undertaken using the Quality Assessment Tool for Quantitative studies [18]. Case reports will be included as part of screening for all available evidence; however, they are inherently at high risk of bias and this will be considered during the assessment of the quality of overall evidence.

The risk of bias in the performance of deep learning models will be evaluated using the QUADAS-2 (Quality Assessment for Diagnostic Accuracy Studies) tool [19]. This will examine the process of patient selection and the conduction and interpretation of the index test and reference standard. An overall risk of bias will be subsequently allocated (high, low, or unclear).

### Data analysis

The two independent reviewers will explore the heterogeneity between the studies using the Review Manager 5.3 provided by the Cochrane Collaboration (1). Potential sources of heterogeneity include the deep learning software, its intention (diagnosis or treatment), the treatment indication and population. A narrative review will be carried out structured around the intervention and outcome of interest. A quantitative analysis (meta-analysis) will be performed if sufficient homogeneous studies in terms of design and outcomes measures are identified.

Statistical heterogeneity will be assessed using the *I*^2^ statistic [20]. A random-effects model will be employed for heterogenous cohorts (*I*^2^ > 50%). The quality of overall evidence will be assessed using The Grading of Recommendations Assessment, Development and Evaluation (GRADE) approach [21]. Sensitivity analysis will be attempted based on the study quality. This may be repeated after removal of poor-quality studies that may affect the overall effect estimate.

## Discussion

Due to the incredible potential of machine learning to process vast amounts of patient information and provide clinically relevant predictions, it is important for plastic surgeons to be informed with the up-to-date applications of this technology in the specialty. The aim of our review is to systematically evaluate the current evidence of this technology in the clinical setting and to discuss the future prospects of machine learning in guiding patient management. To the authors’ knowledge, this is the first systematic review to evaluate the applications of artificial intelligence in plastic surgery.

## Supplementary information

**Additional file 1.**

## Data Availability

The datasets generated and/or analysed during the current study are available in the MEDLINE (OVID SP), EMBASE (OVID SP), Science Citation Index, ClinicalTrials.gov and CENTRAL repositories. https://ovidsp.ovid.com/ http://mjl.clarivate.com/cgi-bin/jrnlst/jloptions.cgi?PC=K https://clinicaltrials.gov/ https://www.cochranelibrary.com/central/about-central

## Abbreviations

CENTRAL: Cochrane Central Register of Controlled Trials
EMBASE: Excerpta Medica Database
GRADE: Grading of Recommendations Assessment, Development and Evaluation
PRISMA-P: Preferred Reporting Items for Systematic Review and Meta-Analysis Protocols
QUADAS-2: Quality Assessment for Diagnostic Accuracy Studies
ROBINS-I: Risk of Bias in Non-randomised Studies-of Interventions
